# Microneedling with Dermaroller

**DOI:** 10.4103/0974-2077.58529

**Published:** 2009

**Authors:** Satish Doddaballapur

**Affiliations:** *Department of Dermatology, Sagar Hospitals, Bangalore, Karnataka, India*

**Keywords:** Dermaroller, microneedling, scars

## Abstract

Microneedling with dermaroller is a new treatment modality for the treatment of scars, especially acne scars, stretch marks, wrinkles, and for facial rejuvenation. It is a simple and relatively cheap modality that also can be used for transdermal drug delivery.

## INTRODUCTION

Dermaroller has recently attained popularity as a simple means of treating scars, particularly acne scars. It can be used safely in a dermatologist's clinic by any dermatologist with minimum training. This article describes salient features of this modality.

## HISTORY

Important milestones in the development of microneedling are as follows:

- 1995-Orentreich and Orentreich described subcision or dermal needling for scars[1]
- 1997-Camirand and Doucet described needle dermabrasion using a "tattoo pistol" to treat scars[2]
- 2006-Fernandes developed percutaneous collagen induction therapy with the dermaroller[3]

## DERMAROLLER-THE INSTRUMENT

The standard dermaroller used for acne scars is a drum-shaped roller studded with 192 fine microneedles in eight rows, 0.5-1.5 mm in length and 0.1 mm in diameter. The microneedles are synthesized by reactive ion etching techniques on silicon or medical-grade stainless steel. The instrument is presterilized by gamma irradiation. Medical dermarollers are for single use only.

## THE PRINCIPLE-COLLAGEN INDUCTION THERAPY

The medical dermaroller needles are 0.5-1.5 mm in length. During treatment, the needles pierce the stratum corneum and create microconduits (holes) without damaging the epidermis. It has been shown that rolling with a dermaroller (192 needles, 200 µm length and 70 µm diameter) over an area for 15 times will result in approximately 250 holes/ cm^2^ . Microneedling leads to the release of growth factors which stimulate the formation of new collagen (natural collagen) and elastin in the papillary dermis. In addition, new capillaries are formed-this neovascularisation and neocollagenesis following treatment leads to reduction of scars.[4‐6] The procedure is therefore aptly called "percutaneous collagen induction therapy" and has also been used in the treatment of photoageing.

## DERMAROLLER FOR ACNE SCARS-THE PROCEDURE

Microneedling is a simple office-based procedure. The area to be treated is anesthetized with topical anesthesia for 45 minutes to one hour. After preparation of the area, rolling is done 15-20 times in horizontal, vertical, and oblique directions; Petechiae or pin-point bleeding which occurs is easily controlled. After treatment, the area is wetted with saline pads. The entire procedure lasts for 15 to 20 minutes, depending on the extent of the area to be treated.

A minimum of six weeks is recommended between two treatments as it takes that long for new natural collagen to form. Three to four treatments may be needed for moderate acne scars.

## POST-PROCEDURE CARE

Microneedling is well tolerated by patients but erythema may be seen after treatment, lasting for 2-3 days. Photoprotection for a week is advised as a routine and local antibiotic creams may be prescribed. The patients can go back to work the very next day.

Apart from erythema, no other side effects have been reported. As the microholes close immediately, postoperative infections do not occur. The procedure is well tolerated and well accepted by the patients, is cost-effective, can be done on all skin types and on areas not suitable for peeling or laser resurfacing, such as near eyes. Microneedling with dermaroller can be combined with other acne scar treatments like subcision, chemical peels, microdermabrasion, and fractional resurfacing, thus maximizing the benefits to the patients.

## HOME-CARE DERMAROLLERS AND DERMA STAMP

Home-Care dermarollers less than 0.15 mm in length are available for transdermal delivery of substances like lipopeptides and other anti-ageing products. They can be used twice a week for up to one hundred times. After use, the rollers have to be cleaned in hot tap water and shaken dry. Peptide-based roller cleansers are available.

Miniature versions of the dermaroller called *dermastamps* have been developed. They are used for localized scars, eg. varicella scars and their needles are 2 mm in length with a diameter of only 0.12 mm. The procedure with the derma-stamp can be performed in two minutes.

## PRACTICAL TIPS

1. Use good quality instruments—there are many instruments from different companies; using poor instruments may lead to breakage of needles in the skin.
2. Counsel the patient that multiple sessions may be needed.
3. Other treatments such as subcision, punch elevation may need to be combined for optimal results in acne scars.
4. Application of EMLA cream anesthesia can prevent procedure pain and help in performing the procedure properly.
5. Allow an interval of 4-6 weeks between the procedures to get good results.
